# Absolute risks of cervical precancer among women who fulfill exiting guidelines based on HPV and cytology cotesting

**DOI:** 10.1002/ijc.32268

**Published:** 2019-04-08

**Authors:** Rebecca Landy, Mark Schiffman, Peter D. Sasieni, Li C. Cheung, Hormuzd A. Katki, Greg Rydzak, Nicolas Wentzensen, Nancy E. Poitras, Thomas Lorey, Walter K. Kinney, Philip E. Castle

**Affiliations:** ^1^ Centre for Cancer Prevention Wolfson Institute of Preventive Medicine, Queen Mary University of London London United Kingdom; ^2^ Division of Cancer Epidemiology and Genetics National Cancer Institute, National Institutes of Health, DHHS Bethesda MD USA; ^3^ Faculty of Life Sciences & Medicine School of Cancer & Pharmaceutical Sciences, Guys Cancer Centre, Guys Hospital, King's College London London SE1 9RT United Kingdom; ^4^ Information Management Services Inc. Calverton MD USA; ^5^ Regional Laboratory Kaiser Permanente Northern California Berkeley CA USA; ^6^ Division of Gynaecologic Oncology Kaiser Permanente Medical Care Program Oakland CA USA; ^7^ Albert Einstein College of Medicine Bronx NY USA

**Keywords:** cervical screening, exiting, cervical cancer, guidelines, HPV, cotesting

## Abstract

US guidelines recommend that most women older than 65 years cease cervical screening after two consecutive negative cotests (concurrent HPV and cytology tests) in the previous 10 years, with one in the last 5 years. However, this recommendation was based on expert opinion and modeling rather than empirical data on cancer risk. We therefore estimated the 5‐year risks of cervical precancer (cervical intraepithelial neoplasia grade 3 or adenocarcinoma *in situ* [CIN3]) after one, two and three negative cotests among 346,760 women aged 55–64 years undergoing routine cotesting at Kaiser Permanente Northern California (2003–2015). Women with a history of excisional treatment or CIN2+ were excluded. No woman with one or more negative cotests was diagnosed with cancer during follow‐up. Five‐year risks of CIN3 after one, two, and three consecutive negative cotests were 0.034% (95% CI: 0.023%–0.046%), 0.041% (95% CI: 0.007%–0.076%) and 0.016% (95% CI: 0.000%–0.052%), respectively (*p*_trend_ < 0.001). These risks did not appreciably differ by a positive cotest result prior to the one, two or three negative cotest(s). Since CIN3 risks after one or more negative cotests were significantly below a proposed 0.12% CIN3+ risk threshold for a 5‐year screening interval, a longer screening interval in these women is justified. However, the choice of how many negative cotests provide sufficient safety against invasive cancer over a woman's remaining life represents a value judgment based on the harms *versus* benefits of continued screening. Ideally, this guideline should be informed by longer‐term follow‐up given that exiting is a long‐term decision.

## Abstract 2

**What's new?**

US guidelines recommend that women older than 65 years cease screening for cervical cancer following two consecutive negative cotests (HPV and cytology) in the previous ten years. However, these guidelines are based on modelling and expert opinion, rather than empirical data. In this large epidemiological study, the authors estimated the risks of cervical precancer following one, two, or three negative cotests among women aged 55‐64 years. They caution that the optimal time for women to exit screening represents a value judgment, based on the harms *versus* benefits of continued screening. Further data are needed.

## Background

Cervical screening is widely acknowledged to be extremely effective at preventing cervical cancer. Current United States (US) and European screening guidelines are based on strong scientific evidence for who should be eligible for screening, which screening test to use (cytology and/or HPV tests), how to manage abnormal screening results and the appropriate length of screening intervals.^1^, ^2^, ^3^, ^4^, ^5^, ^6^, ^7^, ^8^, ^9^, ^10^ However, there is very little evidence available on the appropriate upper age limit to exit women from routine screening. Among countries with established screening programs, the upper age limit for cervical screening is inconsistent, varying from age 60 years in Finland, the Netherlands and Ireland^11^, ^12^ to age 69 years and older in Australia, Japan, Norway and Uruguay.^11^, ^13^, ^14^, ^15^

Until recently, some of the screening recommendations in the US did not have an upper age limit,^16^ and cervical screening over age 65 years was common. In 2012, the US cervical screening guidelines were revamped, recommending concurrent HPV and cytology testing (known as cotesting) every 5 years or cytology testing every 3 years for women aged 21–65 years.^17^ These guidelines recommended that women exit routine cervical screening at age >65 years if they have at least three consecutive negative cytology tests or two negative cotests since age 55 years, with no CIN2+ (cervical intraepithelial neoplasia grade 2 or worse) in the last 20 years, and that the most recent screen occurred within the past 5 years. The 2018 recommendation statement for the US Preventive Services Task Force (USPSTF) cervical screening guidelines maintained the 2012 guidelines for women exiting cervical screening.^18^ In practice, with a 5‐year interval for cotesting, as in the 2018 recommendations, a woman's last screening test could occur 5 years prior to the recommended exiting age.

However, recommendations for exiting criteria are based only on expert opinion and mathematical modeling because of the lack of empirical data. The US consensus recommendation was labeled as “weak” due to the lack of empirical data,^17^ and the choice of age 65 years to cease screening was acknowledged to be based solely on expert opinion. This led to a call for prospective studies in older women as a key research priority.^17^ Furthermore, there is no empirical evidence underlying the requirement for two negative cotests, with no reported data on the risk of precancerous lesions or cancer after two negative cotests compared to risks after one or three negative cotests among women who would be eligible to exit screening.

Data from Kaiser Permanente Northern California (KPNC) represents the largest and longest experience with cotesting in the world. KPNC clinical guidelines differ slightly from the national guidelines given above. Although national guidelines currently recommend cotesting every 5 years, KPNC has recommended cotesting every 3 years since its introduction in 2003. The updated 2012 consensus guidelines for the management of abnormal screening tests recommended a 3‐year screening interval after an HPV‐negative atypical squamous cells of undetermined significance (ASCUS) cotest result.^19^ The 2013 KPNC clinical guidelines also recommend a woman with an HPV‐negative ASCUS cotest result should have their next screen 3 years later, although in KPNC this corresponds to a return to routine screening.^19^ The updated 2012 consensus guidelines explicitly state that a woman should not be exited from screening with an HPV‐negative ASCUS result.^19^ Since 2014, KPNC guidelines have recommended exiting women aged 66 years and older whose most recent cotest since age 55 years was negative, or who had three negative cytology tests at least a year apart since age 55 years, provided they did not have prior CIN2+.^20^

We calculate short‐term (3‐ and 5‐year) risks of cervical intraepithelial neoplasia (CIN) grade 3 and adenocarcinoma *in situ* (AIS) (CIN3) as well as CIN grade 2, CIN3 and AIS (CIN2+) after one, two and three negative cotests among 346,760 women aged 55–64 years; no woman was diagnosed with cancer after one or more negative cotests in this subcohort. These estimates of short‐term risks provide information on how much precancer would be present if a further screening test were to occur at the same interval. We also calculate the frequency of abnormal screening test results after negative cotests.

## Methods

We analyzed prospectively collected data from women whose healthcare was provided by Kaiser Permanente Northern California (KPNC). The dataset contains all cervical screening tests (both HPV and cytology) and results which took place between January 1, 2003, and December 31, 2015, as well as colposcopy and biopsy data between these dates. Across all ages, there are over 1.4 million women with at least one record in this dataset. The cohort has been described in detail previously.^21^ The KPNC Institutional Review Board approved use of the data, and the National Institutes of Health Office of Human Subjects Research deemed this study exempt from IRB review.

Our inclusion and exclusion criteria were as follows. We identified women aged 55–64 years who had at least one negative cotest, with at least one subsequent screen. For this analysis, we considered HPV‐negative ASCUS as a positive cotest, in line with the updated 2012 consensus guidelines.^19^ As women who had negative cotests close together may have been on more intensive follow‐up due to an earlier abnormal result which we do not have a record of, when identifying women who had two and three consecutive negative cotests, we restricted this to negative cotests which were at least 18 months apart. We defined the date of the *n*th consecutive negative cotest as *T*_n_. Although women whose second consecutive negative cotest was before age 60 years would not be eligible to be exited according to national guidelines, we believe that the results from women with two negative cotests aged 55–59 years would be a good approximation for women aged 60–64 years. Women with stand‐alone cytology or HPV tests between the two or three negative cotests were excluded from the analyses. In addition, we excluded cotests taken within a week of a biopsy, as these were unlikely to be screening tests. We excluded all screening or colposcopy data after a hysterectomy or excisional treatment, as well as women diagnosed with CIN2+ prior to the interval of interest.

We categorized the data according to screening rounds, in order to determine what disease was diagnosed as a result of performing one additional round of screening. We defined a screening round to continue until a woman was no longer recommended to have more intensive follow‐up due to an earlier abnormal result, according to the 2012 consensus guidelines for the management of abnormal cervical cancer screening tests and cancer precursors.^19^ Details on how a screening round was defined can be seen in Supporting Information Material 1.

It is important to evaluate the harms as well as the benefits of screening. Since the number of colposcopies is often considered as a surrogate of the main harms (overtreatment and complications) of screening older women,^17^ we tabulated the number and proportion of women whose screening results should lead to a colposcopy referral (two consecutive HPV‐positive tests, any high‐grade cytology [cancer, high‐grade squamous intraepithelial lesion (HSIL), atypical squamous cells cannot rule out HSIL (ASC‐H) or atypical glandular cells (AGC)], an HPV‐positive ASCUS or low‐grade squamous intraepithelial lesion [LSIL] cotest, or two consecutive ASCUS or LSIL cytology tests), as well as the number and proportion of women known to have attended colposcopy in the screening round after *T*_n_, and the number and proportion diagnosed with CIN3 and CIN2+. We considered CIN3 to be the best proxy for cervical cancer risk, however, CIN2 has historically been treated. We therefore also present results for CIN2+ risk. Additionally, when the number of CIN3 diagnoses was very low, we used CIN2+ as the outcome. To see whether there were clinically significant differences in these risks, which would lead us to draw different conclusions based on these variables, we also tabulated these results by age at *T*_n_ (55–59 years, 60–64 years; when considering one or two consecutive negative cotests [very few women have three negative cotests aged 55–59 years]), the time between the last two negative cotests (1.5–2.5 years, 2.5–3.5 years and 3.5+ years, when considering two or three consecutive negative cotests), and the time between *T*_n_ and the following screening test (<1.5 years, 1.5–2.5 years, 2.5–3.5 years and 3.5+ years).

### Statistical methods

Since we do not know exactly when screen‐detected disease occurred, only the date at which it was diagnosed, we considered the date at which disease became detectable to be “interval censored”; that is, we know that it occurred between two dates. To estimate the absolute risk of CIN3 and CIN2+ after one, two and three negative cotests, we used the Turnbull algorithm,^22^ a nonparametric method of analysis for interval‐censored data. We assumed that all disease diagnosed after *T*_n_ was not present at *T*_n_. Ninety‐five percent confidence intervals (95% CI) for the Turnbull absolute risk estimates were estimated through bootstrapping, using 1000 bootstrap resamples. We considered the start of the interval in which disease could have occurred (i.e., the last time we are confident that disease was not present) to be the latest date of (*i*) a second (or subsequent) consecutive negative cotest, or (*ii*) a third (or subsequent) consecutive negative cytology or cotest prior to diagnosis, and the end of the interval to be the date of the biopsy which resulted in a diagnosis. Women without a diagnosis contributed data to the risk estimates after *n* negative cotests provided they had at least one screening test after the *n*th negative cotest. These women were right‐censored (i.e., had no upper bound on when they developed disease). When considering risks after two or three negative cotests, the start of the interval was the latest date at which a second consecutive negative cotest or third consecutive negative cytology/cotest occurred. When considering risks after a single negative cotest, the start of the interval was the latest date at which a single negative cotest occurred, or a negative cytology test after a negative cotest. A test for trend was carried out, using a weighted generalized linear model. We additionally estimated the 3‐ and 5‐year risks of CIN3 and CIN2+ after a negative cotest which followed a positive cotest (both including and excluding HPV‐negative ASCUS as a positive cotest), when both screens were taken at ages 55–64 years. We present absolute risks, since it is the absolute risk of future disease which is important when considering exiting women from cervical screening.

Women whose last recorded screening result was an unresolved positive result (i.e., a positive screening result, which should have led to a colposcopy referral or more intensive screening, and had not yet returned to routine screening) are at higher risk of CIN2+ than women whose last recorded result was negative, though in the analyses described above both were right‐censored and treated in the same way. We therefore present results with and without adjustment for unresolved positive screening results. Details of the adjustment are in Supporting Information Material 2.

Since current US screening guidelines recommend exiting after two negative cotests, regardless of previous screening results, we examined how the risk of CIN2+ being diagnosed during the screening round after one, two and three consecutive negative cotests was influenced by the previous screening test result. We tabulated the following screening test result, stratified by the preceding screening test result, even if the preceding test was taken before age 55 years. We split the result of the screening test preceding the negative cotest(s) by all combinations of HPV (negative, positive or not available) and cytology (negative for intraepithelial lesions or malignancy [NILM], ASCUS, LSIL, high‐grade or not available) results. For stratified analyses in which there were insufficient numbers of CIN3 diagnoses, CIN2+ was used as the primary outcome.

Analyses were carried out in Stata v14^23^ and R v3.5.^24^

## Results

There were 346,760 women aged 55–64 years with at least one screening or biopsy record. After exclusions, 174,205 women had a single negative cotest with at least a single screening test after this cotest; the corresponding numbers for women with two and three consecutive negative cotests were 63,813 and 10,549, respectively. The proportion of women with an unresolved positive screening result was similar after one (1.2%), two (1.3%) and three (1.4%) negative cotests. The majority of women were aged 55–59 years at their first negative test (78.0%) and 60–64 years at their second (62.1%) and third (97.2%) negative cotest (Table 1). Most women with two negative cotests had 2.5–3.5 years between their negative cotests (70.9%), and 69.8% of women with three negative cotests had 2.3–3.5 years between their second and third negative cotests. Median lengths of total follow‐up after one, two and three negative cotests were 3.8 years (IQR 3.0–6.1 years, maximum 12.4 years), 3.1 years (IQR 3.0–3.8 years, maximum 10.6 years) and 3.0 years (IQR 2.6–3.2 years, maximum 8.6 years), respectively.

**Table 1:** The number of women who attend colposcopy, are diagnosed with CIN3 and diagnosed with CIN2+ after one, two and three consecutive negative cotests at age 55–64 years by age at the last negative cotest, time between negative cotests and time between the last negative cotest and the after cotest, adjusted for incomplete follow up

	One negative cotest						Two consecutive negative cotests						Three consecutive negative cotests					
	Women		Women diagnosed with CIN3^1^		Women diagnosed with CIN2+^1^		Women		Women diagnosed with CIN3^1^		Women diagnosed with CIN2+^1^		Women		Women diagnosed with CIN3^1^		Women diagnosed with CIN2+^1^	
	*n*	%	*n*	%	*n*	%	*n*	%	*n*	%	*n*	%	*n*	%	*n*	%	*n*	%
Overall	174,205	100	47.2	0.027	148.9	0.085	63,813	100	5.8	0.009	28.4	0.044	10,549	100	2.7	0.025	2.7	0.025
Age at first/second/third negative cotest (Years)																		
55–59	135,922	78.0	37.8	0.028	115.9	0.085	24,177	37.9	1.5	0.006	11.8	0.049	298	2.8	0.0	0.000	0.0	0.000
60–64	38,283	22.0	9.5	0.025	33.0	0.086	39,636	62.1	4.3	0.011	16.3	0.041	10,251	97.2	2.1	0.020	2.1	0.020
Interval between *T*_n_ and *T*_n − 1_ (Years)																		
1.5 to <2.5							12,918	20.2	2.7	0.021	7.2	0.056	2,956	28.0	0.0	0.000	0.0	0.000
2.5 to <3.5							45,216	70.9	2.8	0.006	19.8	0.044	7,359	69.8	1.8	0.025	1.8	0.025
≥3.5							5,679	8.9	0.0	0.000	0.0	0.000	234	2.2	0.0	0.000	0.0	0.000
Interval between *T*_n + 1_ and *T*_n_ (Years)																		
<1.5	7,005	4.0	1.1	0.016	8.7	0.124	1,576	2.5	0.0	0.000	0.0	0.000	416	3.9	0.0	0.000	0.0	0.000
1.5 to <2.5	23,871	13.7	6.8	0.028	25.4	0.107	8,327	13.0	0.0	0.000	4.0	0.048	2,289	21.7	1.3	0.058	1.3	0.058
2.5 to <3.5	116,108	66.7	34.2	0.029	91.6	0.079	47,768	74.9	4.0	0.008	21.3	0.045	7,148	67.8	0.0	0.000	0.0	0.000
≥3.5	27,221	15.6	5.9	0.022	22.4	0.082	6,142	9.6	1.5	0.024	3.0	0.049	696	6.6	0.0	0.000	0.0	0.000

Assuming women with unresolved positive results are missing at random given their positive result, see Supporting Information Material 2 for details.

1 No women were diagnosed with invasive cervical cancer.

Risks of having an abnormal screening result after one, two and three negative cotests were 3.2, 2.5 and 2.3%, respectively (Table 2), with 2.1, 1.6 and 1.4% of women having abnormal cytology, and 1.8, 1.5 and 1.3% testing HPV positive. The same proportion of women had high‐grade cytology after one, two and three negative cotests (0.2%). The proportion of negative cotests was very similar for women whose second negative cotest was at age 55–59 years (97.4%) and 60–64 years (97.5%), as was the proportion of positive HPV tests (1.5% in each age group; data not shown).

**Table 2:** Cotest results after one, two and three negative cotests

	After 1 negative cotest						After 2 negative cotests						After 3 negative cotests					
Cytology result	HPV negative		HPV positive		Total		HPV negative		HPV positive		Total		HPV negative		HPV positive		Total	
	*n*	%	*n*	%	*n*	%	*n*	%	*n*	%	*n*	%	*n*	%	*n*	%	*n*	%
NILM	168,557	98.6	2032	63.9	170,589	97.9	62,202	99.0	596	61.9	62,798	98.4	10,310	99.0	88	63.3	10,398	98.6
ASCUS	2027	1.2	646	20.3	2,673	1.5	515	0.8	201	20.9	716	1.1	76	0.7	30	21.6	106	1.0
LSIL	139	0.1	394	12.4	533	0.3	45	0.1	130	13.5	175	0.3	8	0.1	17	12.2	25	0.2
High‐grade	300	0.2	110	3.5	410	0.2	88	0.1	36	3.7	124	0.2	16	0.2	4	2.9	20	0.2
Total	171,023	100.0	3,182	100.0	174,205	100.0	62,850	100.0	963	100.0	63,813	100.0	10,410	100.0	139	100.0	10,549	100.0

Abbreviation: NILM, negative for intraepithelial lesions or malignancy; ASCUS, atypical squamous cells of undetermined significance; LSIL, low‐grade squamous intraepithelial lesion.

We present the proportion of women diagnosed with CIN3 and CIN2+ in the following screening round, and absolute risks of CIN3 and CIN2+ at 3 and 5 years with adjustment for unresolved positive screening results as the primary results; the crude results without this adjustment are shown in Supporting Information Tables S1 and S2.

Overall, 1.34, 1.03 and 0.92% of women would have qualified for referral to colposcopy based on their screening results after one, two and three negative cotests. With adjustment for unresolved positive screening results, 0.027% (1 in 3,963), 0.009% (1 in 10,998) and 0.025% (1 in 3,956) of women were diagnosed with CIN3 in the screening round after one, two and three negative cotests aged 55–64 years (Table 1). The corresponding percentages for CIN2+ were 0.085% (1 in 1,170), 0.044% (1 in 2,248) and 0.025% (1 in 3,956) of women, respectively. No women were diagnosed with cancer for the duration of follow‐up available. The adjustment for unresolved positive screening results had a large impact on risks after three consecutive negative cotests; there was a relative increase of 167%, heavily influenced by five women with unresolved high‐grade cytology on their screening test after three negative cotests. Using only observed data, without adjustment for unresolved positive screening results, 69, 170 and 89 women attended colposcopy per CIN3 diagnosed and 22, 32 and 89 per CIN2+ diagnosed after one, two and three negative cotests, respectively.

### Absolute risks

Table 3 shows the absolute risks of CIN3 and CIN2+ at 3 and 5 years after one, two and three negative cotests. Three‐ and five‐year risks of CIN3 after one negative cotest were 0.025% (95% CI: 0.014–0.036%; 1 CIN3 in 4,000 women) and 0.034% (95% CI: 0.023–0.046%; 1 CIN3 in 1,941 women), respectively. By comparison, 3‐ and 5‐year risks of CIN3 after two negative cotests were 0.010% (95% CI: 0.000–0.025%; 1 in 10,296) and 0.041% (95% CI: 0.007–0.076%; 1 in 2,420), respectively. Three‐ and 5‐year risks of CIN3 after three negative cotests were both 0.016% (95% CI: 0.000–0.052%; 1 in 6,250).

**Table 3:** Three‐ and five‐year absolute risks of CIN3 and CIN2+, after one, two and three negative cotests, adjusted for unresolved positive results

	*n*	3‐year risk of CIN3		5‐year risk of CIN3		3‐year risk of CIN2+		5‐year risk of CIN2+	
		Absolute risk	95% CI	Absolute risk	95% CI	Absolute risk	95% CI	Absolute risk	95% CI
One negative cotest	174,205	0.025%	(0.014, 0.036)	0.034%	(0.023, 0.046)	0.061%	(0.037, 0.085)	0.128%	(0.101, 0.155)
Two negative cotests	63,813	0.010%	(0.000, 0.025)	0.041%	(0.007, 0.076)	0.040%	(0.019, 0.062)	0.092%	(0.045, 0.139)
Three negative cotests^1^	10,549	0.016%	(0.000, 0.052)	0.016%	(0.000, 0.052)	0.016%	(0.000, 0.052)	0.016%	(0.000, 0.052)

Assuming women with unresolved positive results are missing at random given their positive result, see Supporting Information Material 2 for details. Confidence intervals are based on bootstrapping. No women were diagnosed with invasive cervical cancer.

1 Based on one observed event.

There was a significant negative trend in 5‐year CIN3 risk with increasing numbers of negative cotests (*p* < 0.001). When stratifying risks after one and two negative cotests by the age of the first/second negative cotest, risks were generally slightly higher for the older women (Supporting Information Table S3). Similar patterns were observed for risks of CIN2+.

Table 4 shows 3‐ and 5‐year risks after a positive cotest (including [*n* = 6,365] and excluding [*n* = 3,456] HPV‐negative ASCUS as a positive cotest) after a negative cotest. The 5‐year CIN3 risks were 0.019% (95% CI: 0.000–0.056%) and 0.038% (95% CI: 0.000–0.108%), respectively. The 5‐year risk of CIN3 including HPV‐negative ASCUS as a positive cotest was significantly lower (*p* < 0.001) than after two negative cotests (0.041%, 95% CI: 0.007–0.076%), and there was no significant difference (*p* = 0.682) when HPV‐negative ASCUS was not considered to be a positive cotest.

**Table 4:** Three‐ and five‐year absolute risks of CIN3 and CIN2+, after one positive cotest followed by a negative cotest, adjusted for unresolved positive results

	*n*	3‐year risk of CIN3		5‐year risk of CIN3		3‐year risk of CIN2+		5‐year risk of CIN2+	
		Absolute risk	95% CI	Absolute risk	95% CI	Absolute risk	95% CI	Absolute risk	95% CI
1 positive (including HPV‐negative ASCUS) followed by 1 negative cotest	6,365	0.019%	(0.000, 0.056)	0.019%	(0.000, 0.056)	0.043%	(0.000, 0.110)	0.176%	(0.000, 0.363)
1 positive (excluding HPV‐negative ASCUS) followed by 1 negative cotest	3,456	0.038%	(0.000, 0.108)	0.038%	(0.000, 0.108)	0.067%	(0.000, 0.166)	0.175%	(0.000, 0.392)

Assuming women with unresolved positive results are missing at random given their positive result, see Supporting Information Material 2 for details. Confidence intervals are based on bootstrapping. No women were diagnosed with invasive cervical cancer.

### Comparing risks after one, two and three negative cotests by the screening result prior to the negative cotests

The result of the previous screening test made a greater difference after one negative cotest than after two or three negative cotests (Table 5); women who had tested HPV positive at the previous screen were much more likely to be diagnosed with CIN2+ after a single negative cotest than women who tested HPV negative (0.519% *vs*. 0.060%, *p* < 0.01). Similarly, women who had abnormal cytology were more likely to be diagnosed with CIN2+ in the screening round after one negative cotest than women with negative cytology (0.255% *vs*. 0.070%, *p* < 0.01). After two negative cotests, women who had previously tested HPV positive were more likely to have CIN2+ diagnosed than women who had not, though this difference was not statistically significant (0.168% *vs*. 0.054%, *p* = 0.25). Women who had abnormal cytology had a slightly higher, though nonsignificant, risk of being diagnosed with CIN2+ compared to women with negative cytology (0.081% *vs*. 0.042%, *p* = 0.52). Compared to women who were HPV negative with abnormal cytology at the previous screening round, women who tested HPV‐positive, cytology‐negative had a nonsignificantly higher risk of CIN2+ (0.206% *vs*. 0.100%, *p* = 0.60). Only one woman was diagnosed with CIN2+ (in fact CIN3) after three negative cotests; her antecedent cotest was negative (i.e., four consecutive negative cotests).

**Table 5:** The number of women who had each cotest result prior to one, two and three negative cotests, and the number and percentage of women diagnosed with CIN2+ by the prior cotest result

Cytology result at test preceding one negative cotest	*n*				CIN2+ (*n*)				CIN2+ (%)			
	HPV result at test preceding one negative cotest											
	HPV negative	N/a*	HPV positive	Total	HPV negative	N/a*	HPV positive	Total	HPV negative	N/a*	HPV positive	Total
NILM	58,166	22,035	799	81,000	33	19	5	57	0.057	0.086	0.626	0.070
N/a*	1,431	–	5	1,436	2	–	0	2	0.140		0.000	0.139
ASCUS	274	106	372	752	0	0	2	2	0.000	0.000	0.538	0.266
LSIL	74	8	125	207	1	0	0	1	1.351	0.000	0.000	0.483
High‐grade	159	9	49	217	0	0	0	0	0.000	0.000	0.000	0.000
Total	60,104	22,158	1,350	83,612	36	19	7	62	0.060	0.086	0.519	0.074
ASCUS+	507	123	546	1,176	1	0	2	3	0.197	0.000	0.366	0.255

Cytology result at test preceding two negative cotests	HPV result at test preceding two negative cotests											
	HPV negative	N/a*	HPV positive	Total	HPV negative	N/a*	HPV positive	Total	HPV negative	N/a*	HPV positive	Total
NILM	18,858	9,210	486	28,554	9	2	1	12	0.048	0.022	0.206	0.042
N/a*	597	–	12	609	1		0	1	0.168		0.000	0.164
ASCUS	925	121	75	1,121	1	0	0	1	0.108	0.000	0.000	0.089
LSIL	35	11	16	62	0	0	0	0	0.000	0.000	0.000	0.000
High‐grade	37	3	7	47	0	0	0	0	0.000	0.000	0.000	0.000
Total	20,452	9,345	596	30,393	11	2	1	14	0.054	0.021	0.168	0.046
ASCUS+	997	135	98	1,230	1	0	0	1	0.100	0.000	0.000	0.081

Cytology result at test preceding three negative cotests	HPV result at test preceding three negative cotests											
	HPV negative	N/a*	HPV positive	Total	HPV negative	N/a*	HPV positive	Total	HPV negative	N/a*	HPV positive	Total
NILM	2,434	2011	69	4,514	1	0	0	1	0.041	0.000	0.000	0.022
N/a*	58		2	60	0		0	0	0.000		0.000	0.000
ASCUS	89	27	2	118	0	0	0	0	0.000	0.000	0.000	0.000
LSIL	3	1	1	5	0	0	0	0	0.000	0.000	0.000	0.000
High‐grade	6	0	0	6	0	0	0	0	0.000			0.000
Total	2,590	2039	74	4,703	1	0	0	1	0.039	0.000	0.000	0.021
ASCUS+	98	28	3	129	0	0	0	0	0.000	0.000	0.000	0.000

Abbreviations: NILM, negative for intraepithelial lesions or malignancy; ASCUS, atypical squamous cells of undetermined significance; LSIL, low‐grade squamous intraepithelial lesion.

*This test was not carried out.

## Discussion

There has been no empirical evidence on which to base exiting guidelines for cervical cancer in the era of HPV testing. In this article, we provide evidence on the absolute risks of CIN3 among women eligible for exiting in the era of cotesting. We have shown that the 5‐year absolute risk of CIN3 after two negative cotests among women aged 55–64 years is less than 1 in 2400, far less than the risk after annual cytology tests, which has been proposed as the risk threshold for 5‐year return (0.12% [1 in 862] in unpublished KPNC data).^25^ The decision to discontinue cervical screening and at what age and risk is a societal one; Swedish guidelines require a single negative HPV test at age 64 years or older,^26^ and Australian guidelines require a single negative HPV test at age 70–74 years.^27^ Still, it must be recognized that it is impractical and very cost ineffective to achieve zero lifetime risk of cervical cancer, even if women have been previously vaccinated against HPV.^28^ However, these results suggest that, at a minimum, a longer screening interval may be appropriate for these low‐risk, older women.

If we consider only the 5‐year risk, then under the principle of “equal management of equal risk,”^1^ we would not screen these women 5 years after even a single negative cotest at age 55–64 years. While 5‐year risk is very low, a woman should only be exited from screening when she is considered to be at sufficiently low risk of cancer for the rest of her life that the harms of further screening outweigh the cancer‐prevention benefits of continuing to screen. However, there has not been sufficient time since the introduction of cotesting to observe long‐term risks of cervical cancer for women exited with negative cotests or HPV tests. To create consistent screening guidelines, ideally an explicit maximum tolerable lifetime cancer risk threshold at which a woman would be exited from cervical screening would be defined. Empirical data would inform the age and screening history that achieves a risk that is less than that threshold.

The most appropriate outcome for determining exiting criteria is the lifetime risk of frank invasive cervical cancer. Asymptomatic lesions such as CIN2 or CIN3, or even early stage asymptomatic cancers, are not a concern if they do not affect a woman's quality of life or life expectancy. No women in our study were diagnosed with cervical cancer. However, since cancer is so rare, and when precancerous lesions are treated, it is often necessary to use precancerous lesions as the outcome to ensure sufficient power.

There is an ongoing debate over the effectiveness of screening in older women.^29^, ^30^ If screening tests or colposcopy were ineffective at screening older women, there would be no advantage to extending the exiting criteria to an older age, even if disease prevalence was sufficiently high to warrant population‐level screening. Although the focus of cervical screening is to detect and treat precancerous lesions, it also detects cancers at earlier stages. Since cancers in older women are diagnosed at more advanced stages^31^ and around 20% of cancers diagnosed aged 65 years or older are in women who exited screening according to guidelines,^32^, ^33^ there could be an advantage of continuing screening to improve the stage distribution (downstage) in cancers among women aged 65 years and older and thereby reduce their morbidity and mortality.

Although there was a statistically significant negative trend in 5‐year CIN3 risk with increasing numbers of negative cotests, the sample size was large and the absolute risk differences may not be clinically significant. It is important to consider harms as well as benefits of screening. A surrogate for the harm of screening older women is the number of colposcopies performed; over 1% of women attended colposcopy in the screening round after two negative cotests, representing 32 colposcopy visits per woman diagnosed with CIN2+. This compares to 22 and 89 colposcopy visits per CIN2+ diagnosed after one and three negative cotests.

It is possible that the birth‐cohort of women currently approaching age 65 years have different risks of cervical cancer to women 10 years older.^34^ For example, the incidence of sexually transmitted gonorrhea peaked in 1975, when women currently aged 60–64 years were aged 18–22 years.^35^ Thus, it is likely that exposure to HPV, a sexually transmitted infection, was also higher in these women compared to women from older birth cohorts. However, if it is reasonable to assume that risks of women exiting over the next 10 years are similar to risks in the birth‐cohort of women who have fulfilled the exiting criteria in the past few years, we at least can use past data to evaluate short‐term risks for women who meet the exiting criteria. It is unlikely that there will be empirical data on the lifetime cancer risk of women being exited at the time the exiting decision is being made. Despite this, further evaluation of the data when more follow‐up time has accrued will enable longer‐term risk estimates to be calculated, and the analysis of women born a few years later once they have also met the exiting criteria will allow us to identify how these risks are changing with time. It is important to regularly review risks among women who have met the exiting criteria, so any cohorts at an increased risk can be identified swiftly, and if appropriate, offered additional screening.^32^ Future work could also consider whether it would be sensible to offer the exiting screening test at a fixed age (e.g., 65 years), rather than after a set interval.

There are limitations when using observed clinical practice data to estimate absolute risks. Since women must attend colposcopy to be diagnosed with CIN2 or CIN3, which are asymptomatic, restricting the analyses to women with a diagnosis of CIN2+ will likely underestimate the absolute risk, due to women with positive screening results not attending colposcopy, despite their (relatively) high risk of CIN2+. While the proportion of women in the study with an unresolved positive screen was low (1.2–1.4%), when considering that only 2.5% of women had an abnormal screening result in the screening round after two negative cotests, a large proportion of the women at highest risk of CIN2+ have not had their disease status verified or been returned to routine screening. This leads to underestimation of the true risk. While we have adjusted for this in the majority of the analyses, the true risks are unknown. We have assumed that the underlying disease status of women with unresolved positive screening tests was missing at random,^36^ given their positive screening result (i.e., that they are the same as women who had the same positive screening result, that was resolved). We were not able to adjust for this when stratifying the risk of CIN2+ by the screening result prior to the negative cotest(s), due to small numbers within each cell.

We do not know why each screening test was taken. This is particularly relevant for screens that took place after a woman had fulfilled the exiting criteria. In theory, women who had fulfilled the exiting criteria would not have any more screening tests, and would therefore not contribute any data to our study unless they had symptomatic testing. These women may be at higher risk than women who fulfilled the exiting criteria and had no subsequent tests, therefore not contributing data to our analyses.

Although there were over 170,000 women with a screening test after a single negative cotest, only 10,000 women had a screening test after three negative cotests, of whom only one woman was diagnosed with CIN2+ (in fact CIN3, 2.1 years after the third negative cotest). There were also limited follow‐up data available for the women who had three negative cotests, as the women needed to have at least four rounds of screening, which are recommended to take place 3 years apart, and only follow‐up after the third negative cotest is considered among these women. We therefore only provide risk estimates at 3‐ and 5‐years, whereas it may be appropriate for a final screening test to take place after a longer interval. There was no additional information on CIN2+ risk after two negative cotests in the screening result prior to the negative cotests, implying that there is no benefit of using screening results from more than the two previous screening rounds when deciding whether to exit a woman.

The results presented here are from a single US Integrated Health System; one which recommends four‐quadrant biopsies as standard. Thus, the sensitivity of colposcopy is likely to be higher than in other screening programs. As we focus on outcomes in a complete screening round after two negative cotests, the sensitivity of colposcopy may change the amount of disease found initially, however since women with a negative colposcopy are recommended to attend further screening at 12 months (which is considered to be part of the same screening round), we assume that (even in settings with less sensitive colposcopy) a second colposcopy would identify the majority of any disease that was missed at the initial colposcopy. We also note that this estimate applies to a low‐risk cohort; these women have private health insurance and have been offered three‐yearly cotesting since 2003, and have no record of a previous CIN2+ diagnosis or excisional treatment. Although they were excluded from our study, as they are not eligible to be exited, no one in KPNC was diagnosed with CIN2+ after one, two or three negative cotests which occurred after their first treatment, though numbers were small (1,146, 410 and 109 women with one, two and three negative cotests aged 55 years and older after treatment, respectively).

No exiting criteria can guarantee absolute safety against cervical cancer. However, it is also not reasonable or feasible to keep women in the screening program when their risk of future disease is too low. We have shown that the 5‐year risks of CIN3 after one, two and three negative cotests aged 55–64 years are all very low in this cohort, implying that at a minimum, a longer screening interval is appropriate. Even with longer follow‐up and an explicit maximum tolerable lifetime cancer risk, no exiting guideline will be 100% safe. The lack of certainty underlying the exiting criteria should be acknowledged in the guidelines.

## Supporting information


**Appendix S1**: Supporting Information
