# Supplementary material for: Absolute risks of cervical precancer among women who fulfill exiting guidelines based on HPV and cytology cotesting
Source: Int J Cancer. 2019 Apr 8;146(3):617–26. doi: 10.1002/ijc.32268 (PMC6742586; doi:10.1002/ijc.32268)
Supplement: Supplementary file 1 — Appendix S1: Supporting Information [file IJC-146-617-s001.docx]

Supplementary Material 1: Defining a screening interval

The next round of screening was defined to begin after the following sets of screening results: 1) a single negative screening test if there were no previous abnormal tests recorded; 2) two consecutive negative screening tests after a positive HPV test (with ≤LSIL cytology, if part of a cotest) or ASCUS/LSIL cytology result, which could have been followed by a colposcopy with ≤CIN1 detected; or 3) three consecutive negative screening tests after a ≥ASCH cytology result which was followed by a colposcopy with ≤CIN1 detected (1). The negative screening tests could be cytology alone, HPV alone, or cotests, provided there were no abnormal results. A cotest was considered negative if the HPV test was negative and cytology result was NILM (Negative for Intraepithelial Lesion or Malignancy). Additionally, we restrict a screening round to screening tests which occurred not more than 6 months after the recommended intervals given in Table 1 (1). We consider colposcopies not taken within 6 months of the referring screening test to be a new screening round.

Table 1: A test is not considered to be part of the same screening round if the time to event is more than 6 months longer than listed below:

| Cytology | HPV test | Recommendation | Subsequent result | Subsequent recommendation |
| --- | --- | --- | --- | --- |
| NILM | negative | 5 years return |  |  |
|  | n/a | 3 years return |  |  |
|  | positive | 1 year return | Positive screen | Colposcopy |
|  |  |  | Negative screen | 3 years return |
| ASCUS | negative | 3 years return |  |  |
|  | n/a | 1 year return | Positive | Colposcopy |
|  |  |  | Negative | 3 years return |
|  | positive | Colposcopy | ≤CIN1 | 1 year return, then  3 year return |
| LSIL | negative | 1 year return | Positive | Colposcopy |
|  |  |  | Negative | 3 years return |
|  | n/a | Colposcopy | ≤CIN1 | 1 year return, then  3 year return |
|  | positive | Colposcopy | ≤CIN1 | 1 year return, then  3 year return |
| ASCH/HSIL/AGC-NOS |  | Colposcopy | ≤CIN1 | 1 year return, then  1 year return, then  3 year return |
| AGC favor neoplasia/AIS |  | Colposcopy | ≤CIN1 | diagnostic excisional procedure |

* Colposcopies are always intended to occur ‘immediately’

Supplementary Material 2: Adjusting for women with an incomplete screening round

In standard interval-censored analyses, all right-censored data are treated the same. However in our scenario we have informative missingness, since women who are censored following an abnormal screening result, but did not attend a subsequent colposcopy which is required to obtain a diagnosis of CIN2 or CIN3, are at higher risk of CIN2+ than women censored following a negative screening result.

We therefore aimed to estimate the amount of missed CIN2+/CIN3+ that would have been detected in the first screening round following two (or one, or three) negative cotests, had every woman completed that screening round. We tabulated the results of the first and second screening tests in the screening round following the two negative cotests, separately for women with and without a complete screening round. If a woman only attended one screening test, the second was recorded as missing. We then tabulated the same data for women with a complete screening round who were diagnosed with CIN2+/CIN3+. For each combination of first and second screening results, we multiplied the proportion of women with complete screening round data who were diagnosed with CIN2+/CIN3+ by the number of women with those test results who had incomplete data for that screening round, and summed across each pair of first and second screening results. This was then repeated, stratified by each of age at the second negative cotest, time between the two negative cotests and time between the second negative cotest and the following cotest. This assumes that having an incomplete screening round occurs at random, given the results of the first two screening tests in that screening round.

To adjust the 3- and 5-year estimates of CIN2+/CIN3+ for women with incomplete screening rounds, we considered the worst screening result among women with complete follow up, and the result prior to CIN2+/CIN3+ diagnosis. The mean time to diagnosis with CIN2+ was 42 days among women diagnosed following 2 negative cotests, and for CIN3+ was 41 days. We assigned each woman a probability of a CIN2+/CIN3+ diagnosis one month after her last recorded result, with the probabilities conditional on the last screening result, and created 10 imputed datasets. The point estimate for the 3- or 5-year risks was the mean risk calculated from the Turnbull analysis on each of the 10 imputed datasets. To calculate the variance of the risk estimate, we combined the within and between-imputation variances using Rubin’s rules (2). The within-imputation variance was calculated from 1000 bootstraps of the first imputed dataset.

1. Massad LS, Einstein MH, Huh WK, Katki HA, Kinney WK, Schiffman M, et al. 2012 updated consensus guidelines for the management of abnormal cervical cancer screening tests and cancer precursors. Obstetrics & Gynecology. 2013;121(4):829-46.

2. Rubin DB. Multiple imputation for nonresponse in surveys: John Wiley & Sons; 2004.

**Supplementary Tables**

Table S1: The number of women who attend colposcopy, are diagnosed with CIN3 and diagnosed with CIN2+ following one, two and three consecutive negative cotests at age 55-64 years by age at the last negative cotest, time between negative cotests and time between the last negative cotest and the following cotest, with no adjustment for unresolved positive screening tests.

|  |  | Women | | Women referred to colposcopy | | Women who attended colposcopy | | Women diagnosed with CIN3 * | | Women diagnosed with CIN2+ * | |
| --- | --- | --- | --- | --- | --- | --- | --- | --- | --- | --- | --- |
|  |  | N | % | N | % | N | % | N | % | N | % |
|  |  | One Negative Cotest | | | | | | | | | |
| Overall | | 174,205 | 100 | 2,340 | 1.34 | 2,421 | 1.39 | 35 | 0.020 | 108 | 0.062 |
| Age at first/second/third negative cotest (Years) | |  |  |  |  |  |  |  |  |  |  |
|  | 55-59 | 135,922 | 78.0 | 1889 | 1.39 | 1905 | 1.40 | 28 | 0.021 | 83 | 0.061 |
|  | 60-64 | 38,283 | 22.0 | 451 | 1.18 | 516 | 1.35 | 7 | 0.018 | 25 | 0.065 |
| Interval between t_n+1_ and t_n_ (Years) | |  |  |  |  |  |  |  |  |  |  |
|  | <1.5 | 7,005 | 4.0 | 132 | 1.88 | 167 | 2.38 | 1 | 0.014 | 8 | 0.114 |
|  | 1.5 to <2.5 | 23,871 | 13.7 | 361 | 1.51 | 412 | 1.73 | 5 | 0.021 | 18 | 0.075 |
|  | 2.5 to <3.5 | 116,108 | 66.7 | 1467 | 1.26 | 1464 | 1.26 | 24 | 0.021 | 64 | 0.055 |
|  | ≥3.5 | 27,221 | 15.6 | 380 | 1.40 | 378 | 1.39 | 5 | 0.018 | 18 | 0.066 |
|  |  | Two Consecutive Negative Cotests | | | | | | | | | |
| Overall | | 63,813 | 100 | 657 | 1.03 | 680 | 1.07 | 4 | 0.006 | 21 | 0.033 |
| Age at first/second/third negative cotest (Years) | |  |  |  |  |  |  |  |  |  |  |
|  | 55-59 | 24,177 | 37.9 | 262 | 1.08 | 273 | 1.13 | 1 | 0.004 | 9 | 0.037 |
|  | 60-64 | 39,636 | 62.1 | 395 | 1.00 | 407 | 1.03 | 3 | 0.008 | 12 | 0.030 |
| Interval between t_n_ and t_n-1_ (Years) | |  |  |  |  |  |  |  |  |  |  |
|  | 1.5 to <2.5 | 12,918 | 20.2 | 146 | 1.13 | 182 | 1.41 | 2 | 0.015 | 6 | 0.046 |
|  | 2.5 to <3.5 | 45,216 | 70.9 | 457 | 1.01 | 454 | 1.00 | 2 | 0.004 | 15 | 0.033 |
|  | ≥3.5 | 5,679 | 8.9 | 54 | 0.95 | 44 | 0.77 | 0 | 0.000 | 0 | 0.000 |
| Interval between t_n+1_ and t_n_ (Years) | |  |  |  |  |  |  |  |  |  |  |
|  | <1.5 | 1,576 | 2.5 | 22 | 1.40 | 27 | 1.71 | 0 | 0.000 | 0 | 0.000 |
|  | 1.5 to <2.5 | 8,327 | 13.0 | 95 | 1.14 | 120 | 1.44 | 0 | 0.000 | 3 | 0.036 |
|  | 2.5 to <3.5 | 47,768 | 74.9 | 474 | 0.99 | 473 | 0.99 | 3 | 0.006 | 16 | 0.033 |
|  | ≥3.5 | 6,142 | 9.6 | 66 | 1.07 | 60 | 0.98 | 1 | 0.016 | 2 | 0.033 |
|  |  | Three Consecutive Negative Cotests | | | | | | | | | |
| Overall | | 10,549 | 100 | 97 | 0.92 | 89 | 0.84 | 1 | 0.009 | 1 | 0.009 |
| Age at first/second/third negative cotest (Years) | |  |  |  |  |  |  |  |  |  |  |
|  | 55-59 | 298 | 2.8 | 2.0 | 0.67 | 1.0 | 0.34 | 0.0 | 0.000 | 0.0 | 0.000 |
|  | 60-64 | 10251 | 97.2 | 95.0 | 0.93 | 88.0 | 0.86 | 1.0 | 0.010 | 1.0 | 0.010 |
| Interval between t_n_ and t_n-1_ (Years) | |  |  |  |  |  |  |  |  |  |  |
|  | 1.5 to <2.5 | 2956 | 28.0 | 22.0 | 0.74 | 20.0 | 0.68 | 0.0 | 0.000 | 0.0 | 0.000 |
|  | 2.5 to <3.5 | 7359 | 69.8 | 71.0 | 0.96 | 66.0 | 0.90 | 1.0 | 0.014 | 1.0 | 0.014 |
|  | ≥3.5 | 234 | 2.2 | 4.0 | 1.71 | 3.0 | 1.28 | 0.0 | 0.000 | 0.0 | 0.000 |
| Interval between t_n+1_ and t_n_ (Years) | |  |  |  |  |  |  |  |  |  |  |
|  | <1.5 | 416 | 3.9 | 6.0 | 1.44 | 8.0 | 1.92 | 0.0 | 0.000 | 0.0 | 0.000 |
|  | 1.5 to <2.5 | 2289 | 21.7 | 16.0 | 0.70 | 18.0 | 0.79 | 1.0 | 0.044 | 1.0 | 0.044 |
|  | 2.5 to <3.5 | 7148 | 67.8 | 71.0 | 0.99 | 60.0 | 0.84 | 0.0 | 0.000 | 0.0 | 0.000 |
|  | ≥3.5 | 696 | 6.6 | 4.0 | 0.57 | 3.0 | 0.43 | 0.0 | 0.000 | 0.0 | 0.000 |

* assuming women with unresolved positive results are missing at random given their positive result, see Supplementary Materials 2 for details

^ No women were diagnosed with invasive cervical cancer

Table S2: 3- and 5-year absolute risks of CIN3 and CIN2+, following one, two and three negative cotests, without adjustment for unresolved positive results^

|  | N | 3-year risk of CIN3 | | 5-year risk of CIN3 | | 3-year risk of CIN2+ | | 5-year risk of CIN2+ | |
| --- | --- | --- | --- | --- | --- | --- | --- | --- | --- |
|  |  | Absolute risk | 95% CI | Absolute risk | 95% CI | Absolute risk | 95% CI | Absolute risk | 95% CI |
| One negative cotest | 174,205 | 0.020% | (0.013, 0.030) | 0.032% | (0.021, 0.042) | 0.049% | (0.038, 0.072) | 0.119% | (0.095, 0.147) |
| Two negative cotests | 63,813 | 0.000% | (0.000, 0.009) | 0.033% | (0.007, 0.075) | 0.030% | (0.011, 0.050) | 0.073% | (0.038, 0.120) |
| Three negative cotests* | 10,549 | 0.011% | (0.000, 0.043) | 0.011% | (0.000, 0.043) | 0.011% | (0.000, 0.043) | 0.011% | (0.000, 0.043) |

Confidence intervals are based on bootstrapping. No women were diagnosed with invasive cervical cancer

*based on one event

^ assuming women with unresolved positive results are missing at random given their positive result, see Supplementary Materials 2 for details

Table S3: 3- and 5-year absolute risks of CIN3 and CIN2+, following one and two negative cotests, stratified by age at the first/second negative cotest, adjusted for unresolved positive results^

|  |  | 3-year risk of CIN3 | | 5-year risk of CIN3 | | 3-year risk of CIN2+ | | 5-year risk of CIN2+ | |
| --- | --- | --- | --- | --- | --- | --- | --- | --- | --- |
| One negative cotest | N | Absolute risk | 95% CI | Absolute risk | 95% CI | Absolute risk | 95% CI | Absolute risk | 95% CI |
| Age 55-59 | 135,922 | 0.027% | (0.015, 0.039) | 0.033% | (0.021, 0.046) | 0.065% | (0.043, 0.087) | 0.118% | (0.090, 0.145) |
| Age 60-64 | 38,283 | 0.023% | (0.006, 0.040) | 0.040% | (0.005, 0.076) | 0.075% | (0.042, 0.107) | 0.183% | (0.093, 0.272) |
| Two negative cotests |  |  |  |  |  |  |  |  |  |
| Age 55-59 | 24,177 | 0.000% | (0.000, 0.023) | 0.023% | (0.000, 0.061) | 0.020% | (0.000, 0.041) | 0.105% | (0.036, 0.168) |
| Age 60-64 | 39,636 | 0.015% | (0.000, 0.031) | 0.058% | (0.006, 0.109) | 0.055% | (0.020, 0.084) | 0.083% | (0.026, 0.138) |

Confidence intervals are based on bootstrapping. No women were diagnosed with invasive cervical cancer

^ Assuming women with unresolved positive results are missing at random given their positive result, see Supplementary Materials 2 for details
